# Flexible Folding: Disulfide-Containing Peptides and Proteins Choose the Pathway Depending on the Environments

**DOI:** 10.3390/molecules26010195

**Published:** 2021-01-02

**Authors:** Kenta Arai, Michio Iwaoka

**Affiliations:** Department of Chemistry, School of Science, Tokai University, Kitakaname, Hiratsuka-shi, Kanagawa 259-1292, Japan; k-arai4470@tokai-u.jp

**Keywords:** protein folding, neurodegenerative diseases, protein engineering, selenoxide, selenopeptides

## Abstract

In the last few decades, development of novel experimental techniques, such as new types of disulfide (SS)-forming reagents and genetic and chemical technologies for synthesizing designed artificial proteins, is opening a new realm of the oxidative folding study where peptides and proteins can be folded under physiologically more relevant conditions. In this review, after a brief overview of the historical and physicochemical background of oxidative protein folding study, recently revealed folding pathways of several representative peptides and proteins are summarized, including those having two, three, or four SS bonds in the native state, as well as those with odd Cys residues or consisting of two peptide chains. Comparison of the updated pathways with those reported in the early years has revealed the flexible nature of the protein folding pathways. The significantly different pathways characterized for hen-egg white lysozyme and bovine milk α-lactalbumin, which belong to the same protein superfamily, suggest that the information of protein folding pathways, not only the native folded structure, is encoded in the amino acid sequence. The application of the flexible pathways of peptides and proteins to the engineering of folded three-dimensional structures is an interesting and important issue in the new realm of the current oxidative protein folding study.

## 1. Introduction

Proteins are synthesized in cells as sequential strings of amino acids connected via peptide bonds. However, the linear peptide chains are in most cases inactive in terms of the biological functions because proteins exert their biological activities only when they are converted into the unique three-dimensional (3D) structures. This structure-forming process of a polypeptide chain is called *protein folding*, and is known to proceed spontaneously in a test tube for many small globular proteins.

Since the pioneering studies by Anfinsen [1,2] and Levinthal [3] in the 1960s, researchers have exerted enormous efforts to elucidate the folding pathways of various proteins. However, it is still not easy to characterize the intermediates, especially when a protein is folded with its disulfide (SS) bonds intact due to the short time constant of the folding reaction (<1 s). On the other hand, an oxidative folding from the SS-reduced state takes place with a much longer time constant (>1 s). Thus, the SS-coupled protein folding is advantageous in detecting the folding intermediates, with which we can delineate a scenario of protein folding.

In early studies of SS-coupled (or oxidative) protein folding, researchers focused on the physicochemical aspects, i.e., observation and characterization of the folding intermediates, although the reactions were frequently monitored under the conditions slightly different in terms of the temperature and pH from those in vivo due to the technical limitations [4]. On the other hand, thanks to the novel experimental techniques developed in the last few decades, the oxidative folding study is moving to a new realm. In short, new types of oxidants (SS-forming reagents), as well as genetic and chemical technologies for synthesizing designed artificial proteins, have been developed. The new oxidants enabled the SS-coupled folding to be performed under biologically more relevant conditions. By applying such new techniques, unprecedented folding pathways have been discovered for several SS-containing proteins, suggesting that folding pathways of proteins are much more flexible than those the researchers proposed previously.

In this review, after a brief overview of the historical and physicochemical aspects of oxidative protein folding study, the recently revised oxidative folding pathways of several representative peptides and proteins (Table 1) are compared with those reported in the early years, focusing on the flexible nature of protein folding pathways. Some possible applications of the flexible folding to engineering the peptide and protein structures are proposed as intriguing issues in the new realm of oxidative protein folding study.

## 2. Historical Background

In 1920–30s, protein denaturation processes were elaborately studied by Wu [5]. Then, about a half century ago, Anfinsen [1,2] proposed a fundamental principle of protein folding, on the basis of the pioneering study using bovine pancreatic ribonuclease A (RNase A) as a model protein; namely, a polypeptide chain eventually folds to one unique structure, called a native state. This principle alternatively brought the researchers a rationale of the so-called Anfinsen’s dogma that the structural information of a protein is encoded in the amino acid sequence. In the meantime, Levinthal [3] theoretically found that, if a polypeptide chain searched for the specific native structure in a trial and error manner, it should require an astronomical time to fold to a protein. Since the protein folding process practically completes within a second for small globular proteins, the big time-scale gap between the theorical prediction and the experimental observation led him to a paradoxical advocation that proteins should fold through their defined pathways. Thus, in an early stage of the protein folding study, the researchers focused on characterizing the folding pathways in order to decode the information of the native-state 3D structure from the amino acid sequence.

In the meantime, it became to be widely recognized in around 1990s that neurodegenerative disorders, such as Creutzfeldt–Jakob disease (CJD), Alzheimer’s disease (AD), Parkinson’s disease (PD), amyotrophic lateral sclerosis (ALS), etc., are related to misfolding of proteins in neurons [6,7], suggesting that the native structure of a protein is not a unique state for the amino acid sequence but one of the possible stable states. Moreover, genetic engineering and chemical peptide synthesis technologies have become available for preparation of designed artificial proteins in the last few decades [8]. With these breakthrough events, the protein folding study is now moving to a new realm where the structures of proteins can be actively controlled. Thus, development of the methods to prevent or cure protein misfolding diseases, as well as fabrication of peptide-based medicines, is not our dream. To reach the goal, a proper understanding of the peptide and protein folding pathways depending on the environment must be especially important [9].

## 3. Physicochemical Aspects of SS-Coupled Protein Folding

To characterize the folding pathways of a protein, it is necessary to observe the transiently forming intermediates during the folding reaction [10]. However, in spite of numerous efforts in the past, observation of the folding intermediates is still not easy due to their inherent conformational flexibility and short lifetime. On the other hand, in the oxidative folding of SS-containing proteins, the reaction generally proceeds on a longer time scale (mins to hours). Indeed, the intermediates (i.e., SS intermediates) generated during the oxidative protein folding can be rather easily characterized by trapping them either by acidification of the reaction solution to pH 2 to 3, which converts the reactive cysteinyl thiolates (S^−^) to essentially unreactive thiols (SH) [11,12], or by chemically capping the S^−^ with quenching reagents, such as iodoacetic acid or iodoacetamide [13]. The SS-intermediates thus inactivated are subsequently separated by HPLC, and their chemical structures, i.e., the SS topologies and conformations, are determined by spectroscopic methods as well as proteolytic digestion. In this way, the oxidative folding pathways of many SS-containing proteins have been proposed.

**Figure 1:**
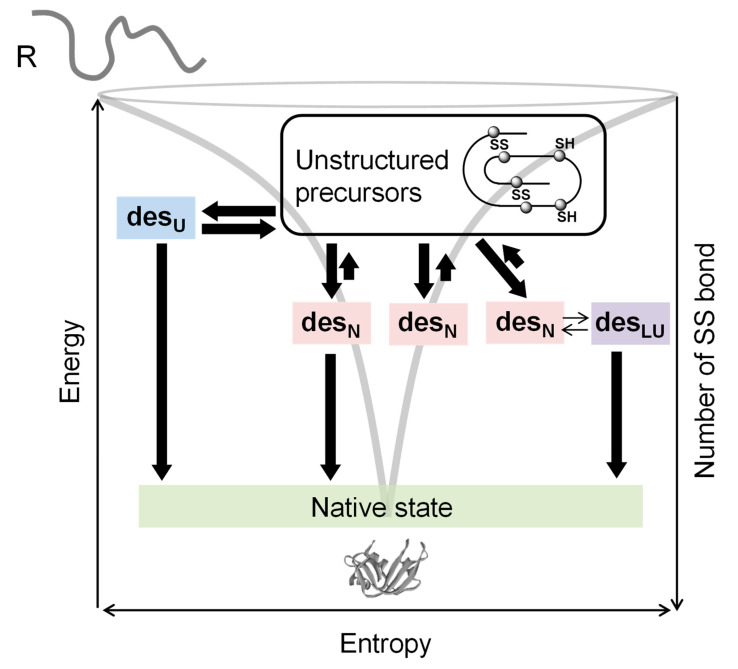
Four typical oxidative folding pathways governed by a folding funnel as the energetic landscape. This figure was made by merging a concept regarding the energetic landscape model by Chang, et al. [4] to the schematic model demonstrated by Scheraga, et al. [14].

Scheraga and co-workers previously categorized the oxidative folding pathways into four typical types (Figure 1) [14]. In each type, a reduced and denatured protein (R) folds through a des intermediate, which is generated by SS rearrangement of partially and randomly oxidized species, i.e., the unstructured precursors (see Figure 1). The des intermediates represent specific SS intermediates lacking one of the native SS bonds present in the native state. The desU has no specific structure, whereas the desN adopts a native-like structure. These des intermediates can be converted to the native state (N) when the direct oxidation to form the last native SS bond is possible. However, some of the desN intermediates cannot directly be oxidized to N due to the stiff conformation. When a local unfolding is possible in the desN to generate desLU, the buried SH groups would gain enough flexibility to form the final SS linkage by oxidation. Chang [4] delineated the relationship between the number of SS bonds and the diversity of conformations (i.e., chain entropy) during the oxidative folding by using a folding funnel as the energetic landscape [15].

**Figure 2:**
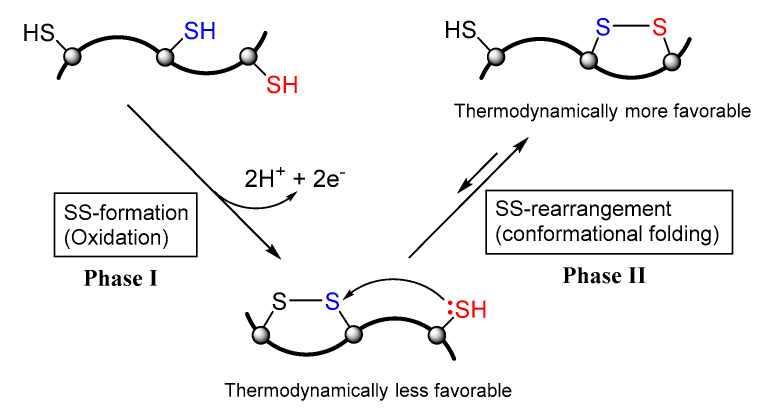
Disulfide formation and reshuffling governed by the thermodynamic stability.

Figure 2 schematically shows the reaction mechanism of SS formation, which is a fundamental process of the oxidative protein folding. To regenerate N from the reduced R, SS bonds must be formed at the correct positions of the native state. When R is oxidized, however, the SS formation usually occurs before the structural folding. Therefore, in this pre-folding event, numerous intermediates having randomly formed SS bonds are generated (Figure 2, Phase I). Then, the partially oxidized species gradually gain the native-like structure accompanied by regeneration of the native SS pairings via SS rearrangement (Figure 2, Phase II). The latter process is governed by thermodynamics of the peptide chain under the applied conditions. Thus, the formation of the native SS bonds after the pre-folding event would be coupled with the conformational shift from the random-coil or collapsed state to the native-like fold [16].

**Scheme 1:**
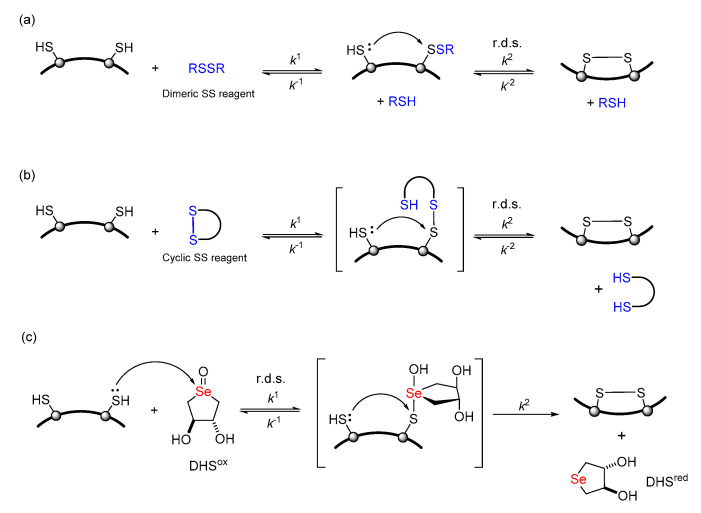
Mechanisms for SS formation using various oxidants. (**a**) A dimeric disulfide (SS) reagent. (**b**) A cyclic SS reagent. (**c**) A selenoxide reagent [19].

In the oxidative folding study, the selection of an oxidant (SS-forming agent) is an important factor to control the pathways of a protein folding because it greatly affects the reaction rate of SS formation, thereby controlling the amounts of SS intermediates that are transiently accumulated in the reaction solution. In early years, aerial oxidation by molecular oxygen (O_2_) was frequently applied, although the oxidation proceeded very slowly with O_2_. To perform the oxidative folding at a reasonable reaction velocity, dimeric SS oxidants, such as glutathione (GSSG), a representative oxidant also in vivo, were subsequently employed. As shown in Scheme 1a, the dimeric SS oxidant induces formation of a SS bond in a protein via a mixed-SS intermediate. However, generation of such SS intermediates was troublesome in analyzing the folding pathways. In this regard, a use of *trans*-4,5-dihydroxy-1,2-dithiane (DTT^ox^) is advantageous because the mixed-SS intermediate would not be populated in the reaction solution (Scheme 1b). This significantly decreases the number of the detectable folding intermediates [17]. However, since oxidation potentials for GSSG and DTT^ox^ (*E*°′ = −256 and −327 mV, respectively [18]) are close to that for cystine (CysSSCys) (*E*°′ = −238 mV [18]), SS formation in a peptide chain should be reversible, hence an excess amount of the oxidant is required to complete the reaction. Moreover, the SS-based oxidants are applicable only under a weakly basic condition (pH 7.5~9.0). As a consequence, clear observation of conformational folding events coupled with SS rearrangement is still not easy even with these SS-based oxidants.

To separate the SS formation and SS rearrangement events during oxidative protein folding, trans-3,4-dihydroxytetrahydroselenophene-1-oxide (DHS^ox^) was developed as a strong SS-forming reagent [19,20], which enables rapid but selective oxidation of the SH groups of a reduced protein molecule (Scheme 1c). The reaction proceeds stoichiometrically in a wide range of pH (i.e., pH 3.0~10.0) through a similar mechanism to that of DTT^ox^. In the first step, the SH group of a peptide chain attacks at the Se atom of DHS^ox^. Then, another SH group of the peptide chain intramolecularly attacks at the S atom of the generated thioselenurane intermediate to produce a SS bond, releasing selenide DHS^red^. Since the oxidation potential of DHS^ox^ (*E*°′ = 375 mV [21]) is remarkably higher than that of cystine, the rate-determining step becomes the first step and the reaction proceeds irreversibly and stoichiometrically. According to these characteristic features of DHS^ox^, the reaction rate of the SS formation was proportional to the number of free SH groups existing in the substrate peptide chain [19,21,22]. Furthermore, the SS formation process completed before the SS reshuffling in the oxidized peptide chain. Thus, Phases I and II of Figure 2 could be separated, making the analysis of the oxidative folding pathways of proteins much easier. In addition to DHS^ox^, inorganic oxidants, such as a platinum complex ([Pt(en)_2_Cl_2_]^2+^), were also employed [23,24,25]. Various diselenide (SeSe)- and SS-based reagents (or catalysts), which can accelerate the oxidative folding velocity, were tested as alternative oxidants (see [26] for a review and [27,28,29,30,31,32,33,34] for recent papers). In the last decade, the enzymes that assist protein folding processes in the endoplasmic reticulum (ER), such as protein disulfide isomerase (PDI), were further applied to enhance both the velocity and yield of oxidative protein folding practically [35].

## 4. Oxidative Folding of Peptides and Proteins

### 4.1. Two-Disulfide Peptides

When a reduced peptide chain (R) having four cysteinyl SH groups is oxidized by a suitable oxidant, a number of heterogeneous SS-intermediates would be generated, including theoretically up to six one-disulfide (1SS) and three two-disulfide (2SS) intermediates. Indeed, sequential formation of the ensembles of 1SS and 2SS intermediates were observed when reduced A-chains of bovine insulin (Ins-A) and human relaxin 2 (Rlx-A) were oxidized with stoichiometric amounts of DHS^ox^ as shown in Equation (1) [21].

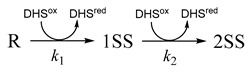
(1)

The kinetic analyses clearly showed that the second order rate constants, *k*_1_ and *k*_2_, which correspond to transformations of R → 1SS and 1SS → 2SS, respectively, were proportional to the number of free SH groups existing along the reactant peptide chains (i.e., *k*_1_: *k*_2_ = 2:1). The observed stochastic SS formation suggested that the SS intermediates of Ins-A and Rlx-A have fully flexible conformations.

**Figure 3:**
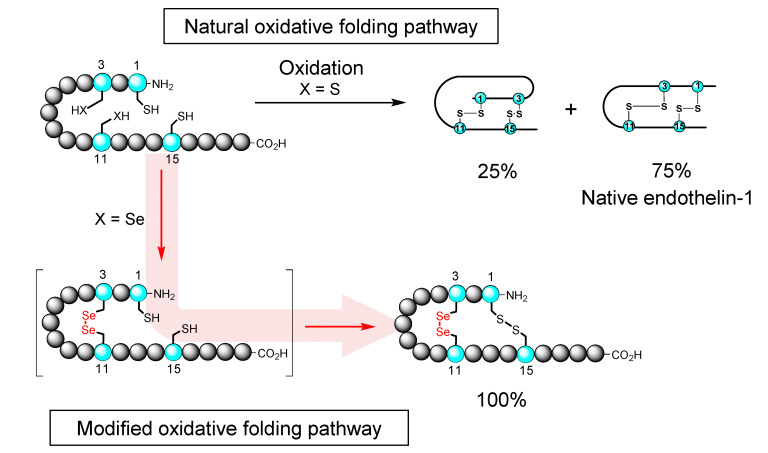
Oxidative folding pathways of [Sec^3^, Sec^11^, Nle^7^]-endothelin-1 (ET-1) via predominant diselenide (SeSe)-bond formation.

On the other hand, when the reduced species of endothelin-1 (ET-1), a vasoconstrictive peptide consisting of 21 amino acid residues, was oxidized with O_2_, only two 2SS products out of three were obtained in a ratio of 1: 3. The minor isomer had non-native SS pairings (i.e., Cys1–Cys11 and Cys3–Cys15), while the other major one had two native SS bonds (i.e., Cys1–Cys15 and Cys3–Cys11) (Figure 3) [36]. Moroder et al. replaced two selenocysteine (Sec) residues for the two inner Cys residues in order to selectively regenerate native ET-1. This SS-to-SeSe strategy relies on the lower reduction potential of a SeSe bond (*E*°′_SeSe_ = −380 ~ −410 mV [37]) than that of a SS bond (*E*°′_SS_ = –230 ~ –260 mV [37]), indicating that a SeSe bond is thermodynamically more stable than a SS bond. Moreover, the p*K*_a_ value of the selenol (SeH) group in Sec is about 3 units lower than that of the SH group. Thus, the SeH group is more reactive than thiols, and, therefore, the formation of a SeSe bond does not compete with the formation of a SS bond, facilitating the peptide chain to fold to the correct structure. In fact, the ET-1 analogue, [Sec^3^, Sec^11^, Nle^7^]-ET-1, selectively produced the SeSe bond, yielding the native folded state exclusively (Figure 3). The obtained SeSe analogue of ET-1 showed almost the same biological activity as native ET-1.

The SS-to-SeSe strategy is useful not only for obtaining correctly folded proteins in high yields but also for steering the folding pathways. Moroder et al. successfully applied the strategy to the oxidative folding of apamin, a small bee venom peptide comprised of 18 amino acid residues having two SS bonds (Cys1–Cys15 and Cys3–Cys11) in the native state. They prepared the all possible isomers, i.e., [Sec1–Sec11, Cys3–Cys15], [Sec3–Sec11, Cys1–Cys15], and [Sec1–Sec3, Cys11–Cys15], and analyzed the relationship between the peptide conformations and the topologies of the SS/SeSe bonds by means of CD and NMR spectroscopy [38]. Similar strategies have been widely applied to various peptides and proteins having multiple SS bond in the native state, such as conotoxine, Ecballium elaterium trypsin inhibitor II (EETI-II), and bovine pancreatic trypsin inhibitor (BPTI) (vide infra) [39,40,41,42,43,44,45].

The success of the SS-to-SeSe strategy demonstrated that the thermodynamic stability of a SeSe bond can change the folding pathways of peptides and proteins. Thus, intrinsically non-productive folding intermediates can be purposely produced and isolated by replacing Sec for the Cys residues.

### 4.2. Three-Disulfide Proteins

**Figure 4:**
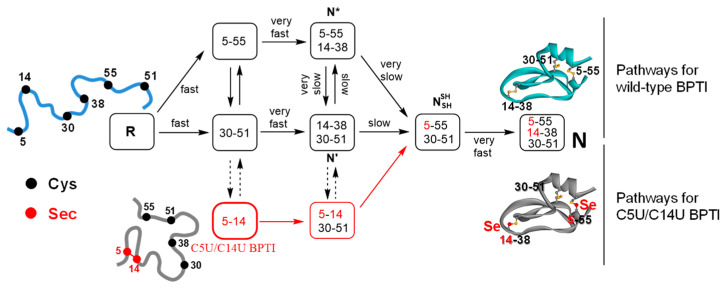
Oxidative folding pathways of wild-type bovine pancreatic trypsin inhibitor (BPTI) (black arrows) and BPTI variant (C5U/C14U) (red arrows).

BPTI is a 58-residue globular protein, having three SS linkages (i.e., Cys5–Cys55, Cys14–Cys38, and Cys30–Cys51) in the native folded state. This small protein was one of the most extensively studied model proteins in the protein folding field before 2000s [46,47,48,49,50,51]. Following the pioneering works of Creighton [46,47], Weissman and Kim [48,49,50] unambiguously determined the oxidative folding pathways as shown with black arrows in Figure 4. All the important SS intermediates were characterized in terms of their SS topologies as well as kinetic and thermodynamic stabilities. In this conventional folding pathways, native BPTI (N) is slowly regenerated from two 2SS intermediates, indicated with symbols N* and N′, which have two native SS bonds between the Cys residues denoted in the boxes. These intermediates are highly structured, thereby being kinetically trapped on the folding pathways.

To detour the sluggish folding pathways of BPTI, Hilvert and Metanis synthesized an artificial (C5U,C14U)-BPTI variant, in which Cys5 and Cys14 are replaced by Sec residues, by applying the solid-phase peptide synthesis methodology [52,53]. The oxidative folding of the obtained variant was indeed accelerated due to appearance of an alternative pathway (Figure 4, red arrows), which goes through the new intermediates having a SeSe bond between Sec5 and Sec14 residues. Thus, the variant can avoid being trapped as N* and N′ and go to $N_{\mathrm{SH}}^{\mathrm{SH}}$ directly. It is of interest that the (C5U,C14U)-BPTI variant does not have a SeSe bond but has two SeS bonds in the native folded state even though a SeS bond is thermodynamically less stable than a SeSe bond. This may reflect the larger conformational stability of N than that of the (Sec5–Sec14, Cys30–Cys51) intermediate.

**Figure 5:**
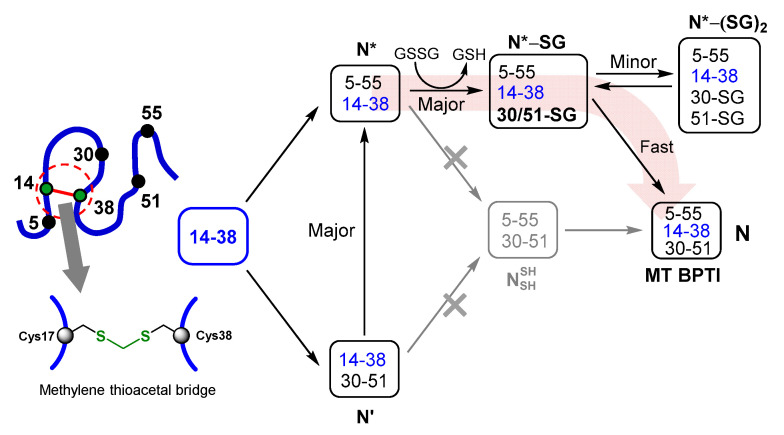
Oxidative folding pathway of MT-BPTI through direct conversion of N* intermediates.

Another interesting attempt to steer the folding pathways of BPTI was recently reported by Metanis’ group [54]. They synthesized a novel BPTI analogue (i.e., MT-BPTI), in which solvent-exposed Cys14 and Cys38 residues are connected via a methylene thioacetal bond (S–CH_2_–S) that cannot be cleaved reductively, in order to arrest the major folding pathways via $N_{\mathrm{SH}}^{\mathrm{SH}}$. Contrary to the expectation that the generation of folded MT-BPTI should be prohibited because $N_{\mathrm{SH}}^{\mathrm{SH}}$, a direct precursor to N, cannot be formed, MT-BPTI folded via a mixed-SS intermediate with GSH (i.e., N*-SG), which would be formed by the reaction of N* with GSSG (Figure 5). Thus, the introduction of the uncleavable SS bond unveiled a new pathway that had been hidden in the previous oxidative folding studies of wild-type BPTI.

**Figure 6:**
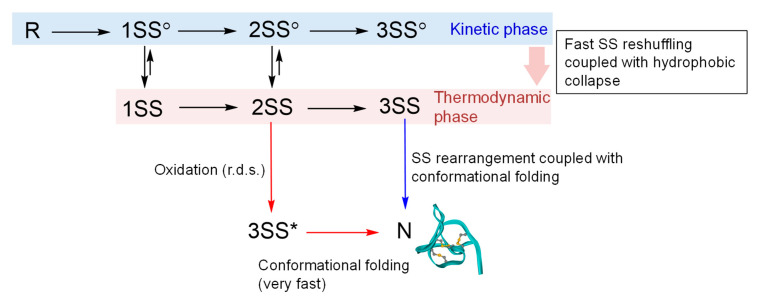
Revised oxidative folding pathways of hirudin, combining the models proposed by Chang (blue arrow) [55,56,57], Scheraga (red arrows) [58], and Arai & Iwaoka [19,21].

Another well-studied small 3SS protein is hirudin, a thrombin inhibitor, that has the SS linkages between Cys6–Cys14, Cys16–Cys28, and Cys22–Cys39. Although the molecular size is similar to BPTI, the oxidative folding pathways of hirudin are in significant contrast to those of BPTI. While only five out of 75 possible SS intermediates were located on the oxidative folding pathways of BPTI (Figure 4, black arrows), more than 30 heterogeneous SS intermediates could be detected for hirudin. Chang [4] reported that ensembles of the scrambled SS intermediates containing one, two, and three SS bonds (1SS, 2SS, and 3SS, respectively) were generated when reduced hirudin (R) was oxidized by O_2_ dissolved in a buffer solution in the presence of β-mercaptoethanol as a thiol-based folding catalyst. This pre-folding process would correspond to a hydrophobic collapse of a random-coil state of the peptide chain to lose the chain entropy. The generated 3SS intermediates were subsequently converted to native hirudin (N) through the intermolecular SS–SH exchange with β-mercaptoethanol in the solution (Figure 6, blue arrow) [55,56,57]). In the meantime, Scheraga et al. reported that when DTT^ox^ was used as an oxidant, the specific 2SS intermediates that had two native SS bonds and were in equilibrium with the other heterogeneous 2SS intermediates, were oxidized to 3SS*, which had three native SS bonds but did not yet get the native structure. The generated 3SS* finally folded to N rapidly with a gain of the conformation stability (Figure 6, red arrows, [58]). Thus, the oxidative folding pathways of hirudin would be switchable by selection of the oxidant employed.

Later on, the early folding events of a recombinant hirudin (CX-397), which has a hybrid sequence of hirudin variants-1 and -3 [59], were reinvestigated using DHS^ox^ as an oxidant [19,21]. The second-order rate constants for R → 1SS, 1SS → 2SS, and 2SS → 3SS (i.e., *k*_1_, *k*_2_, and *k*_3_, respectively) were roughly in a ratio of 3:2:1, being proportional to the number of the SH groups present in the reactant (see Section 3), under acidic to neutral pH conditions (Table 2). However, under weakly basic conditions, the ratio became significantly deviated due to the conformational shift from the stochastically formed SS intermediates (1SS°, 2SS°, and 3SS°) to the thermodynamically stabilized SS-intermediates (1SS, 2SS, and 3SS). In fact, in the presence of guanidinium chloride (Gdn-HCl) as a denaturant, the ratio of 3:2:1 was almost recovered although the overall reaction was decelerated due probably to the interactions between a guanidinium ion and the peptide molecule. Formation of a more compact structure in 3SS than in 3SS° was supported by the fluorescence measurement. Thus, the presence of kinetic and thermodynamic phases in the early SS formation event was elucidated by using DHS^ox^ as a selective and strong oxidant.

Very recently, Shimamoto and Hidaka [60] reported on the folding of a topological isomer of enterotoxin, which is a 3SS short polypeptide produced by enterotoxigenic E. coli. The topological isomer, possessing the three native SS bonds but with a different folded structure, was obtained by regioselective sequential SS-bond formation, while under an aerial oxidation condition only native enterotoxin was generated. This is an interesting example demonstrating that the oxidative folding pathways of a protein are flexible depending on the conditions. The misfolded enterotoxin could be rescued to the native structure in the presence of an external thiol like the case of the 3SS intermediate ensemble of hirudin.

### 4.3. Four-Disulfide Proteins

**Figure 7:**
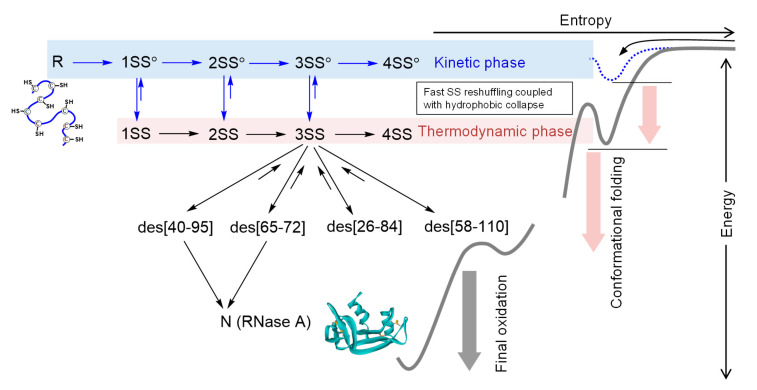
Oxidative folding pathways of RNase A, combining the models proposed by Scheraga, et al. [61,62] and Iwaoka, et al. [63,64].

Bovine pancreatic ribonuclease A (RNase A) has been a symbolic target of biological studies for a long time. This well-characterized globular protein contains four SS linkages, two of which are formed between Cys40–Cys95 and Cys65–Cys72 stabilizing the flexible loop domains and the other two are formed between Cys26–Cys84 and Cys58–Cys110 connecting the α-helix and β-sheet regions in the native state. The oxidative folding pathways of RNase A were well characterized by Scheraga and co-workers using SS-based oxidants, such as DTT^ox^ and GSSG [17,61,62]. In the early folding event, reduced RNase A (R) was sequentially oxidized to SS-intermediate ensembles (i.e., 1SS, 2SS, 3SS, and 4SS) having no specific structure. The generated 3SS intermediates were subsequently rearranged to thermodynamically stable des species, which have three native SS bonds but lack one native SS bond denoted in brackets (Figure 7). This rate-determining SS-rearrangement process occurred with conformational folding of the native-like structure. At 25 °C, only des[40-95] and des[65-72] were observed as the key folding intermediates, which could be oxidized to the native state (N) [61]. On the other hand, des[26-84] and des[58-110], which were observed only at the temperature below 15 °C, were dead-end species and could not be directly oxidized to N [62]. However, characterization of these des intermediates was not easy in the presence of excess amounts of the oxidants.

To monitor the oxidative folding pathways of RNase A more clearly, we applied DHS^ox^ as an alternative oxidant [22]. Interestingly, while a use of DHS^ox^ did not change the oxidative folding pathways, it revealed the presence of kinetic and thermodynamic phases, which corresponds to the stochastic SS formation with a loss of the chain entropy and the rapid SS reshuffling with a gain of the slight stabilization due to the hydrophobic collapse, respectively, in the pre-folding event [63]. In addition, since DHS^ox^ enabled stoichiometric SS formation (Scheme 1c) and no oxidant was left in the reaction solution, the rate-determining steps became the final oxidation processes of des[40-95] and des[65-72] to N, resulting in accumulation of these des species. Hence, the conformational folding processes of RNase A, i.e., 3SS → des intermediates, could be cleanly monitored. After the oxidation of R with DHS^ox^, a pseudo-equilibrium was indeed attained between 3SS and the des intermediates. Therefore, the thermodynamic stability (∆*G*) of the des intermediates could be estimated from the equilibrium constants [64]. Interestingly, while des[40-95] was more stable than des[65-72] at 25 °C (∆∆*G* = −0.7 kcal/mol), the relative stability was inverted at 35 °C (∆∆*G* = 0.4 kcal/mol). Thus, at the temperature close to a physiological condition, des[65-72] would play a more important role than des[40-95] as a key folding intermediate leading to native RNase A.

**Figure 8:**
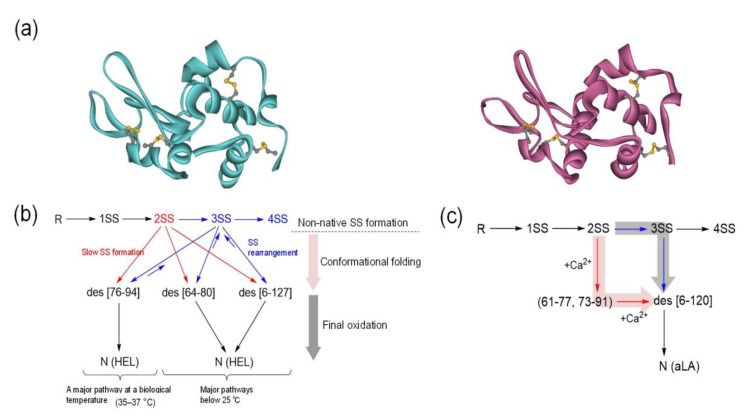
Oxidative folding pathways of hen egg-white lysozyme (HEL) and αLA. (**a**) 3D structures of HEL (left) and αLA (right). (**b**) Oxidative folding pathways of HEL. The red and blue arrows represent pathways proposed by Dobson et al. [65,66] and Iwaoka et al. [67], respectively. (**c**) Oxidative folding pathways of αLA. The red and blue arrows are major pathways in the presence and absence of Ca^2+^, respectively.

A temperature effect on the oxidative folding pathways of a protein was more obvious in the case of hen egg-white lysozyme (HEL) (Figure 8a). Dobson et al. [65] reported that the heterogeneous intermediate ensembles, 1SS and 2SS, with no specific structure were generated from reduced HEL (R) in the early folding event at 20 °C (Figure 8b). Then, the 2SS was slowly oxidized to three des intermediates, des[6-127], des[64-80], and des[76-94], having three native SS linkages. Finally, by oxidation of the two Cys residues left in the des intermediates, native HEL (N) was regenerated. While the oxidation steps of des[64-80] and des[6-127] to N shared the major folding pathways of HEL, the oxidation of des[76-94] was hindered and occurred only with a long reaction time or in the presence of PDI through the other two des intermediates [65,66].

This classical scenario has been revised recently by applying DHS^ox^ as an oxidant [67]. When R was reacted with stoichiometric amounts of DHS^ox^, not only 1SS and 2SS but also 3SS and 4SS were generated in a similar manner that was observed for hirudin and RNase A (see above). Then, the 3SS was gradually converted to the three des intermediates via slow SS rearrangement (Figure 8b). More importantly, des[64-80] and des[6-127], which were located on the major folding pathways at 25 °C and below, were not formed at 35 °C. Instead, only des[76-94] was observed at the temperature, and it was directly oxidized to N by addition of DHS^ox^. Thus, the major folding pathways at the physiological temperature can be invisible at the low temperatures.

Bovine α-lactalbumin (αLA) is another representative four-disulfide protein, for which the oxidative folding pathways were well studied [68]. Although αLA shares a similar native structure as well as a SS topology with HEL, the oxidative folding pathways were essentially different (Figure 8c). Since αLA had a calcium binding pocket in the β-sheet domain, in which two SS linkages of Cys61–Cys77 and Cys73–Cys91 are located, the presence of a calcium ion (Ca^2+^) greatly affected the oxidative folding pathways as well as the folding velocity and the yield of native αLA (N) [69,70,71]. Indeed, under a metal-free condition, SS formation of reduced αLA (R) took place at random and produced heterogeneous SS intermediate ensembles (1SS, 2SS, 3SS, and 4SS), resulting in a low yield of N (a hirudin-like folding). In the presence of Ca^2+^, on the other hand, two structured SS intermediates, which were assigned to (61-77, 73-91) and des[6-120], were rapidly populated (a BPTI-like folding). The (61-77, 73-91) intermediate would form by SS rearrangement of 2SS and can subsequently be oxidized to N through the des[6-120] intermediate. Thus, the oxidative folding pathways of αLA can be controlled by the presence of a metal ion. In this case, however, the folding pathways were robust against the temperature change [71].

### 4.4. Oxidative Folding of Proteins with Odd Cys Residues

With few exceptions, many small globular proteins have an even number of cysteine residues and no free thiol group in the native state. Therefore, the oxidative folding pathways of a protein with odd Cys residues were not well known until recently. Probably the first extensive study of such a case was reported using bovine milk β-lactoglobulin (BLG) as a model protein [72].

**Figure 9:**
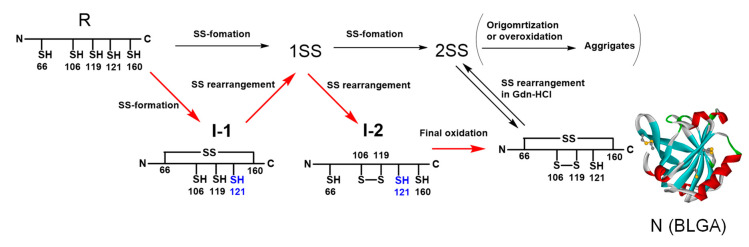
Oxidative folding pathways of bovine milk β-lactoglobulin (BLGA) with odd Cys residues. Red and black arrows represent major and minor pathways, respectively.

BLG is a major whey protein with one free cysteinyl thiol (Cys121) in addition to two SS bonds (Cys66–Cys160 and Cys106–Cys119). While the SS-intact conformational folding had been studied in depth [73], the oxidative folding pathways remained unknown. When reduced BLG variant A (BLGA) (R) was oxidized with DHS^ox^, a limited number of SS intermediates were observed (Figure 9). Among the produced SS intermediates, two distinct 1SS intermediates, which have native Cys66–Cys160 SS bond (I-1) or another native Cys106–Cys119 SS bond (I-2), could be characterized. In the major folding pathway, R, which was rich in α-helices, was firstly oxidized to I-1 and subsequently converted to I-2 via SS rearrangement. Finally, I-2 gained the missing Cys66–Cys160 SS bond by oxidation to regenerate native BLGA (N), which is rich in β-sheets. In addition to this major folding pathway, N could also be regenerated from the scrambled 2SS intermediate ensemble via SS rearrangement, but this minor pathway would be deteriorative because the 2SS was prone to self-aggregate irreversibly probably due to the presence of a free Cys SH group. During the oxidative folding of BLGA, the redundant Cys121 SH group may assist efficient SS-rearrangement of the SS intermediates, guiding them to I-1 and then I-2, not to 2SS [72].

The oxidative folding pathways of odd Cys proteins remain largely unknown and will be interesting targets in the future study.

### 4.5. Oxidative Folding Pathways of Two Chain Proteins

Elucidation of oxidative folding pathways of two chain proteins is another interesting and challenging issue. With this regard, the folding pathway of insulin was recently characterized finally and has been applied to facile chemical synthesis of insulin by a native chain assembly (NCA) method [74].

**Figure 10:**
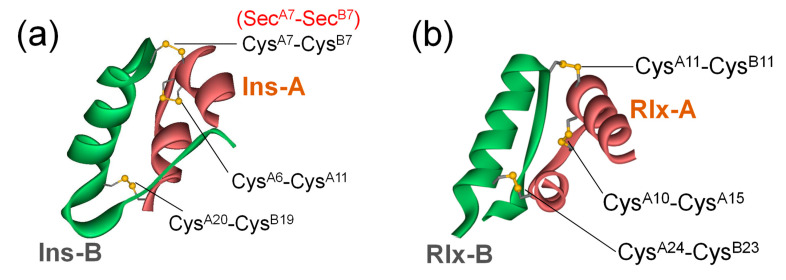
3D structures of insulin and relaxin. (**a**) Bovine pancreatic insulin (BPIns). (**b**) Human relaxin 2 (Rlx).

Insulin is a small globular protein (5.8 kDa), comprised of two peptide chains, i.e., A-chain (Ins-A, 21 amino acid residues) and B-chain (Ins-B, 30 amino-acid residues), which are crosslinked by Cys^A7^-Cys^B7^ and Cys^A20^-Cys^B19^ SS bridges (Figure 10a). Another SS bond, Cys^A6^-Cys^A11^, is located in the A-chain. In pancreatic β-cells, insulin is produced as proinsulin, a single polypeptide chain of 86 amino acid residues, in which the C terminus of the B-chain is linked to the N-terminus of the A-chain by a connecting C-peptide. The proinsulin is effectively oxidized in ER to obtain the three native SS bonds and is then converted to the mature two-chain insulin by proteolytic cleavage of the C-peptide. The oxidative folding pathways of proinsulin in vivo were well characterized by Feng et al. [75,76].

There is a long history of the attempts to artificially prepare insulin and its analogues. The initial attempt was a very simple approach of just mixing the reduced A- and B-chains in a folding buffer solution [77,78,79]. However, this primitive strategy disappointingly resulted in a low yield of insulin (less than 5%). Following this unsuccessful attempt, a variety of attempts, such as an approach of the directed SS crosslinking between the two chains and a biomimetic approach of the proinsulin-like single-chain folding, have been considered to increase the insulin yield. However, none of them has been fabricated to the practical level to supply insulin in the market.

**Figure 11:**
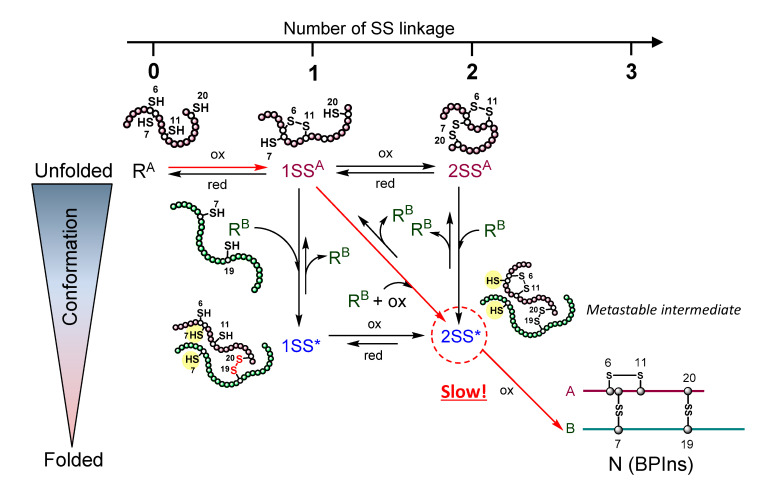
Oxidative two-chain folding pathways of BPIns revealed by the native chain assembly method.

In this context, to know the exact two-chain folding pathways of insulin would be a sober but steady approach. According to this line, we carried out the crosslinking reactions between reduced and non-protected bovine Ins-A and -B chains under various conditions, which included the conditions using DHS^ox^ as an oxidant [74]. The revealed major folding pathways are summarized in Figure 11, where 1SS^A^ and 2SS^A^ represent one- and two-SS intermediate ensembles, respectively, of Ins-A, the major component of which has a native Cys^A6^–Cys^A11^ SS bond. In the folding pathways, 2SS*, a heterodimeric 2SS isomer having two out of three native SS bonds, i.e., Cys^A6^–Cys^A11^ and Cys^A20^–Cys^B19^, is a pivotal precursor of N. The spectroscopic characterization demonstrated that 2SS* has a native-like folded structure, indicating that the 2SS* preferentially converts to N, otherwise it might easily dissociate to 2SS^A^ and R^B^ through intramolecular SS reshuffling. The key 2SS* intermediate would be generated through several paths, such as from 1SS^A^ by the reaction with both R^B^ and an oxidant, from 1SS*, which has a native SS crosslink between Cys^A20^–Cys^B19^, by oxidation, and from 2SS^A^ through the intermolecular SS-SH exchange reaction with R^B^. The revealed two-chain folding pathways of insulin strongly suggest that the yield of insulin by NCA can be increased by stabilizing 2SS* by any methods. Predictably, under the optimized NCA condition (at pH 10.0 and −10 °C), bovine pancreatic insulin (BPIns) and human insulin (HIns) were obtained in up to 39% and 49% yields, respectively. Similarly, human type-II relaxin (Rlx2) (Figure 10b), which is a pregnancy peptide hormone and a representative member of the insulin family, was regenerated in up to 49% by applying the NCA condition [74].

**Figure 12:**
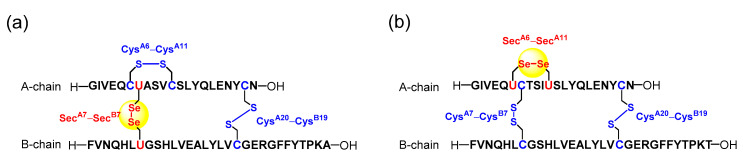
Primary amino acid sequences of the selenoinsulin series. (**a**) Bovine selenoinsulin reported by Arai et al. [80]. (**b**) Human selenoinsulin reported by Metanis et al. [81].

Application of the SS-to-SeSe strategy (see Section 4.1 and Section 4.2) to insulin folding has already been applied by mixing the synthesized Sec-replaced Ins-A and -B chains, i.e., C7U^A^ and C7U^B^, under the NCA condition [80]. The [C7U^A^, C7U^B^] insulin analogue, so-called selenoinsulin (Se-Ins) (Figure 12), was efficiently generated in up to 27% isolated yield even under slightly milder condition (at 4 °C) than that employed for NCA of wild-type insulin. The folding of Se-Ins would be assisted by the spontaneous chain assembly probably via selective formation of the Sec^A7^-Sec^B7^ SeSe crosslink. Thus, the SS-to-SeSe strategy can be an efficient method to modulate the folding pathways of two-chain proteins too.

Metanis and coworkers [81] have also performed a similar chain assembly synthesis of human insulin analogue, i.e., a [C6U^A^, C11U^A^] Se-Ins variant, by reacting the Sec-replaced A-chain with the wild-type B-chain. Both the Se-Ins’s prepared by us [80] and Metanis [81] exhibited a bioactivity comparable to the wild-type insulin, indicating that the molecular structures of Se-Ins are almost the same as the wild-type in solution.

## 5. Flexibility of the Oxidative Folding Pathways

During the oxidation of a polypeptide chain having several Cys residues, two elementary reactions, i.e., SS formation and SS rearrangement, are necessarily involved (Figure 2). These reactions indeed control the oxidative folding pathways to the native state. As seen above for several model peptides and proteins, there seems to be no general rule how to delineate the oxidative folding pathways based on the number and topology of the SS bonds present in the native folded state. This feature is obvious by looking at the significantly different pathways of HEL and αLA (Figure 8), which are classified to the same protein superfamily [82]. Thus, oxidative folding pathways of a protein would depend primarily on the amino acid sequence. This in turn suggests that the information about the folding pathways, not only the 3D folded structure, of a protein would also be encoded in the amino acid sequence.

In the previous stage of protein folding study, oxidative folding pathways were clearly characterized for several representative SS-containing proteins. However, as exemplified above, recent reinvestigation of those pathways applying a new oxidant as well as an artificially designed polypeptide, has disclosed that the folding pathways are significantly flexible depending on the conditions, such as the temperature, pH, and co-existing metal ions. In some cases, the previously proposed pathways may be hindered, or the previously hidden pathways may be possible, under physiologically relevant conditions. Indeed, in the synthesis of a protein in vivo, the polypeptide chain elongated on the ribosome is transported into the endoplasmic reticulum (ER), where the oxidative folding is initiated from the N-terminal domain in the presence of various SS-oxidoreductases and SS-isomerases, such as PDI and its family proteins [83]. Thus, the oxidative folding pathway of a full-length polypeptide characterized in a test tube could be different from that in the ER. The roles of PDI family proteins are especially important because they efficiently promote the oxidative folding by retrieving kinetically trapped intermediates back to the normal pathways and also by avoiding the formation of metastable oligomers or aggregates. This indicates that ER-resident PDI family enzymes and possibly their mimic catalysts as well would be able to control the major oxidative folding pathways in vitro. Thus, an appropriate use of the enzymes and mimics should be a practical strategy to improve the yield and velocity of the oxidative folding that is often a bottleneck process in chemical synthesis of proteins [26,27,28,29,30,31,32,33,34,35].

From the viewpoint of peptide and protein engineering, flexibility of the folding pathways would be fascinating. It would be applicable to control the structures, thereby functions, of proteins by changing the folding conditions. For example, by changing folding conditions on purpose, a polypeptide chain might fold to a new structure through a different pathway. More practically, by applying the recently available new folding strategies, such as a use of a strong and selective oxidant and the substitution of Cys by Sec, the proteins that could not be folded under classical folding conditions would be obtained effectively. Prevention and treatment of protein misfolding diseases would also be possible by controlling the protein folding pathways under well-managed folding conditions.

## 6. Perspectives and Concluding Remarks

In the classical oxidative folding study, the folding reaction was frequently conducted under non-natural environments so that the key SS intermediates could be easily observed. This indeed allowed the researchers to characterize the oxidative folding pathways of some representative proteins and propose a general model of protein folding (Figure 1). At the same time, they gradually noticed that the folding pathways greatly depends on the reaction environments as well as the type of proteins. In this review, recent achievements in the relevant fields are overviewed. It should be emphasized here that the folding pathways of peptides and proteins are significantly flexible.

In relation to prevention and treatment of protein misfolding diseases, it is preferable to investigate the protein folding pathways under the conditions that are close to physiological environments, if possible, at an organ level, where numerous factors coexist. In the meantime, the methods to control the folding pathways are now available by applying the state-of-the-art technologies as illustrated in this review. Engineering peptide and protein structures and the functions by controlling the folding pathways are interesting and important issues in the new realm of the current protein folding study.

## Figures and Tables

**Table 1:** Model peptides and proteins for the oxidative folding study.

Category	Proteins	Number of SS bonds	Number of Cys Residues	SS-Bond Topology	Section
2SS	insulin A-chain	2	4	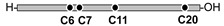	4.1
	relaxin A-chain	2	4	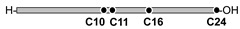	
	endothelin-1	2	4	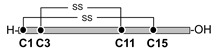	
3SS	BPTI	3	6	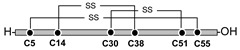	4.2
	hirudin	3	6	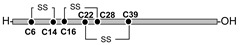	
	enterotoxin	3	6	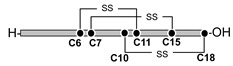	
4SS	ribonuclease A	4	8	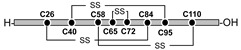	4.3
	lysozyme	4	8	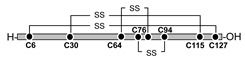	
	α-lactalbumin	4	8	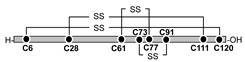	
Odd Cys	β-lactoglobulin	2	5	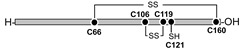	4.4
Tow-chain	insulin selenoinsulin	3 2 + 1SeSe	4/2 *^a^*	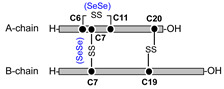	4.5
	relaxin 2	3	4/2 *^a^*	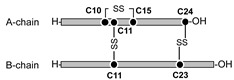	

*^a^* Numbers of Cys (or Sec; selenocysteine) residues in the A/B chains, respectively.

**Table 2:** The second-order rate constants for oxidation (SS formation) of hirudin CX-397 [19,21].

pH and Additive	*k*_1_ for R → 1SS (mM^−1^ s^−1^)	*k*_2_ for 1SS → 2SS (mM^−1^ s^−1^)	*k*_3_ for 2SS → 3SS (mM^−1^ s^−1^)
4.0	4.3 ± 0.2	2.6 ± 0.1	1.2 ± 0.1
7.0	9.8 ± 0.5	6.6 ± 0.3	2.8 ± 0.3
8.0	18.6 ± 1.0	10.0 ± 0.6	3.6 ± 0.6
8.0, +2 M Gdn-HCl	9.8 ± 0.6	5.0 ± 0.3	2.7 ± 0.2
